# World Polio Day — October 24, 2014

**Published:** 2014-10-24

World Polio Day was established for annual observance on October 24 by Rotary International more than a decade ago to commemorate the fight against poliomyelitis. Widespread use of poliovirus vaccine led to an increasing number of polio-free countries and to establishment of the Global Polio Eradication Initiative (GPEI) in 1988. As of October 14, a total of 243 polio cases had been reported in 2014, with 92% of the cases reported from Nigeria, Afghanistan, and Pakistan, the only three countries where transmission of indigenous wild poliovirus has continued uninterrupted (1).

On December 2, 2011, the CDC Emergency Operations Center was activated to strengthen the agency’s partnership engagement through GPEI. In April 2012, the World Health Assembly declared completion of polio eradication a programmatic emergency for global public health (2). In May 2014, the international spread of poliovirus was declared a public health emergency of international concern (3,4). Additional information regarding CDC’s polio eradication activities is available at http://www.cdc.gov/polio/updates, and additional information about GPEI and the global partnership is available at http://www.polioeradication.org.
